# Supplementary material for: The Clinical Effects of Type 2 Diabetes Patient Management Using Digital Healthcare Technology: A Systematic Review and Meta-Analysis
Source: Healthcare (Basel). 2022 Mar 13;10(3):522. doi: 10.3390/healthcare10030522 (PMC8953302; doi:10.3390/healthcare10030522)
Supplement: Supplementary file 1 [file healthcare-10-00522-s001.zip › healthcare-1597641-supplementary.pdf]

Supplementary

Table S1. Search Strategy in PubMed

1	"diabetes mellitus, type 2"[MeSH Terms]
2	(((((((((((((((((((((((((((((((("Diabetes Mellitus, Noninsulin-Dependent"[Text Word]) OR ("Diabetes Mellitus, Ketosis-Resistant"[Text Word])) OR ("Diabetes Mellitus, Ketosis Resistant"[Text Word])) OR ("Ketosis-Resistant Diabetes Mellitus"[Text Word])) OR ("Diabetes Mellitus, Non Insulin Dependent"[Text Word])) OR ("Diabetes Mellitus, Non-Insulin-Dependent"[Text Word])) OR ("Non-Insulin-Dependent Diabetes Mellitus"[Text Word])) OR ("Diabetes Mellitus, Stable"[Text Word])) OR ("Stable Diabetes Mellitus"[Text Word])) OR ("Diabetes Mellitus, Type II"[Text Word])) OR ("NIDDM"[Text Word])) OR ("Diabetes Mellitus, Noninsulin Dependent"[Text Word])) OR ("Diabetes Mellitus, Maturity-Onset"[Text Word])) OR ("Diabetes Mellitus, Maturity Onset"[Text Word])) OR ("Maturity-Onset Diabetes Mellitus"[Text Word])) OR ("Maturity Onset Diabetes Mellitus"[Text Word])) OR ("MODY"[Text Word])) OR ("Diabetes Mellitus, Slow-Onset"[Text Word])) OR ("Diabetes Mellitus, Slow Onset"[Text Word])) OR ("Slow-Onset Diabetes Mellitus"[Text Word])) OR ("Type 2 Diabetes Mellitus"[Text Word])) OR ("Noninsulin-Dependent Diabetes Mellitus"[Text Word])) OR ("Noninsulin Dependent Diabetes Mellitus"[Text Word])) OR ("Maturity-Onset Diabetes"[Text Word])) OR ("Diabetes, Maturity-Onset"[Text Word])) OR ("Maturity Onset Diabetes"[Text Word])) OR ("Type 2 Diabetes"[Text Word])) OR ("Diabetes, Type 2"[Text Word])) OR ("Diabetes Mellitus, Adult-Onset"[Text Word])) OR ("Adult-Onset Diabetes Mellitus"[Text Word])) OR ("Diabetes Mellitus, Adult Onset"[Text Word])) OR ("T2DM"[Text Word])) OR ("T2D"[Text Word])
3	1 OR 2
4	"wearable electronic devices"[MeSH Terms] OR "mobile applications"[MeSH Terms] OR "smartphone"[MeSH Terms]
5	(("App, Mobile"[Text Word]) OR ("App, Mobile"[Text Word])) OR ("App, Portable Electronic"[Text Word])) OR ("App, Portable Software"[Text Word])) OR ("Application, Mobile"[Text Word])) OR ("Application, Portable Electronic"[Text Word])) OR ("Application, Portable Software"[Text Word])) OR ("Applications, Mobile"[Text Word])) OR ("Applications, Portable Electronic"[Text Word])) OR ("Applications, Portable Software"[Text Word])) OR ("Apps, Mobile"[Text Word])) OR ("Apps, Portable Electronic"[Text Word])) OR ("Apps, Portable Software"[Text Word])) OR ("Device, Wearable"[Text Word])) OR ("Device, Wearable Electronic"[Text Word])) OR ("Devices, Wearable"[Text Word])) OR ("Devices, Wearable Electronic"[Text Word])) OR ("digital health apps"[Text Word])) OR ("Electronic Application, Portable"[Text Word])) OR ("Electronic Applications, Portable"[Text Word])) OR ("Electronic Apps, Portable"[Text Word])) OR ("Electronic Device, Wearable"[Text Word])) OR ("Electronic Devices, Wearable"[Text Word])) OR ("Electronic Skin"[Text Word])) OR ("Electronic App, Portable"[Text Word])) OR ("health, mobile"[Text Word])) OR ("mhealth"[Text Word])) OR ("Mobile Application"[Text Word])) OR ("Mobile Apps"[Text Word])) OR ("mobile health"[Text Word])) OR ("mobile intervention"[Text Word])) OR ("mobile technologies"[Text Word])) OR ("mobile technology"[Text Word])) OR ("Mobile App"[Text Word])) OR ("Phones, Smart"[Text Word])) OR ("Portable Electronic App"[Text Word])) OR ("Portable Electronic Application"[Text Word])) OR ("Portable Electronic Applications"[Text Word])) OR ("Portable Electronic Apps"[Text Word])) OR ("Portable Electronic App"[Text Word])) OR ("Portable Software App"[Text Word])) OR ("Portable Software Application"[Text Word])) OR ("Portable Software Applications"[Text Word])) OR ("Portable Software Apps"[Text Word])) OR ("Skin, Electronic"[Text Word])) OR ("smart

	devices"[Text Word])) OR ("Smart Phone"[Text Word])) OR ("Smart Phones"[Text Word])) OR ("smartphone app"[Text Word])) OR ("Smartphones"[Text Word])) OR ("smartwatch"[Text Word])) OR ("Software App, Portable"[Text Word])) OR ("Software Application, Portable"[Text Word])) OR ("Software Applications, Portable"[Text Word])) OR ("Software Apps, Portable"[Text Word])) OR ("Technologies, Wearable"[Text Word])) OR ("Technology, Wearable"[Text Word])) OR ("Wearable Device"[Text Word])) OR ("wearable devices"[Text Word])) OR ("Wearable Electronic Device"[Text Word])) OR ("Wearable Electronic Devices"[Text Word])) OR ("wearable health device"[Text Word])) OR ("wearable health devices"[Text Word])) OR ("wearable system"[Text Word])) OR ("wearable systems"[Text Word])) OR ("Wearable Technologies"[Text Word])) OR ("Wearable Technology"[Text Word])) OR ("wearables"[Text Word])) OR ("wireless devices"[Text Word])) OR ("wireless health"[Text Word])) OR ("wireless technology"[Text Word])) OR ("wearable behavioral intervention"[Text Word])
6	4 OR 5
7	3 AND 6
